# From Entry to Egress: Strategic Exploitation of the Cellular Processes by HIV-1

**DOI:** 10.3389/fmicb.2020.559792

**Published:** 2020-12-04

**Authors:** Pavitra Ramdas, Amit Kumar Sahu, Tarun Mishra, Vipin Bhardwaj, Ajit Chande

**Affiliations:** Molecular Virology Laboratory, Indian Institute of Science Education and Research (IISER) Bhopal, Bhopal, India

**Keywords:** HIV-1 infection, cell organelles, capsid uncoating, restriction factors, host-virus interactions

## Abstract

HIV-1 employs a rich arsenal of viral factors throughout its life cycle and co-opts intracellular trafficking pathways. This exquisitely coordinated process requires precise manipulation of the host microenvironment, most often within defined subcellular compartments. The virus capitalizes on the host by modulating cell-surface proteins and cleverly exploiting nuclear import pathways for post entry events, among other key processes. Successful virus–cell interactions are indeed crucial in determining the extent of infection. By evolving defenses against host restriction factors, while simultaneously exploiting host dependency factors, the life cycle of HIV-1 presents a fascinating montage of an ongoing host–virus arms race. Herein, we provide an overview of how HIV-1 exploits native functions of the host cell and discuss recent findings that fundamentally change our understanding of the post-entry replication events.

## Introduction

Human immunodeficiency virus (HIV)-1 is a complex retrovirus known to infect humans and diminish the immune system leading to acquired immunodeficiency syndrome (AIDS). The virus measures about 100 nm with viral envelope glycoproteins (gp120 and gp41) trimers embedded in the host cell-derived lipid membrane. This envelope encases a conical capsid that contains two copies of an RNA genome (∼9.2 kb) in addition to the retroviral enzymes. The HIV-1 genome encodes accessory proteins (Vif, Vpr, Vpu, and Nef) and regulatory proteins, Tat and Rev, apart from the canonical proteins (Gag, Pol, and Env) that other retroviruses encode. The gag gene translates into a polyprotein comprised of matrix (MA), capsid (CA), and nucleocapsid (NC). The pol gene encodes for the enzymes protease (PR), reverse transcriptase (RT), and integrase (IN). The env gene encodes for the viral surface glycoprotein comprising of surface (SU), gp120 and transmembrane (TM), gp41. In addition to the structural and accessory proteins encoding regions, the genome is flanked by long terminal repeats (LTRs). Since HIV-1 encodes a few functional genes, host cell machinery plays a rather significant role in completing the virus life cycle. Thus, this review provides a conceptual advance on how HIV-1 exploits intracellular processes most required during its journey in and out of the host cell. Providing with an updated model of the viral life-cycle, we also highlight the latest findings that fundamentally change our understanding of post-entry steps.

## Plasma Membrane: The Site of Virion Fusion and Entry

**FIGURE 1:**
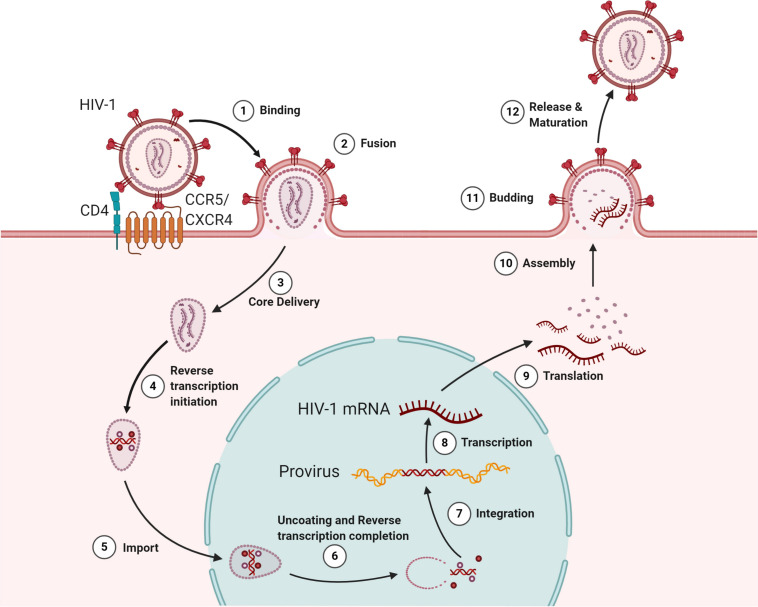
The HIV lifecycle. The infection begins when the envelope glycoprotein attaches to the receptor CD4 and the membrane-spanning co-receptors (CXCR4/CCR5) (step 1), facilitating the entry and fusion of the viral particle into the target cell (step 2). Following core delivery (step 3), reverse transcription begins in the cytoplasm (step 4), and the core is imported into the nucleus (step 5). Following the nuclear import, uncoating and reverse transcription completes (step 6) and viral integrase facilitate viral genome integration into the host chromosome (step 7). Proviral transcription (step 8) yields viral RNAs that are exported to the cytoplasm for viral protein production (step 9). Genome-length viral RNA and viral proteins are assembled to package into virions for budding (steps 10 and 11). Ensuing budding, the virus progeny releases and matures to become an infectious virion (step 12).

**FIGURE 2:**
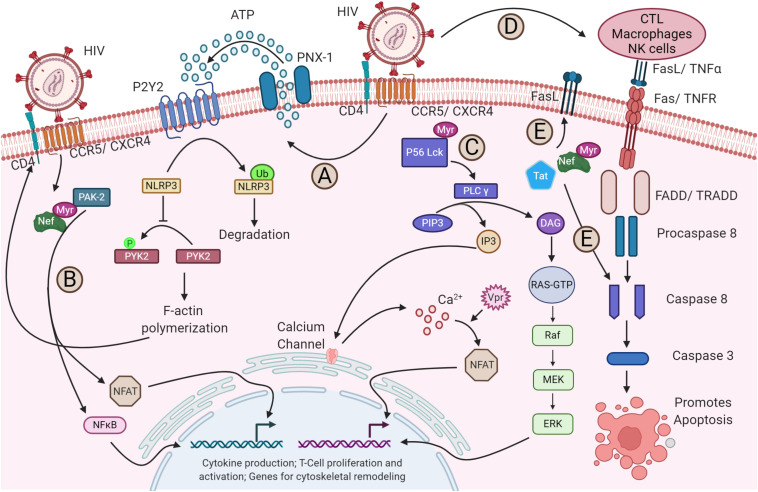
Alteration of host signaling pathways by HIV-1. **(A)** The binding of HIV-1 to its receptor and co-receptor triggers the activation of P2Y2 by releasing ATP from host cytosol through pannexin-1 (PNX-1). The activated P2Y2 promotes the ubiquitin-mediated degradation of NLRP3, facilitating the phosphorylation and activation of PYK-2, which subsequently enables the F-actin polymerization required for the fusion and entry of HIV-1 into the host cell (not shown). **(B)** Nef activates NFAT and NF-κB via PAK2, which triggers the expression of cytoskeletal remodeling genes. **(C)** Binding of the virus also activates the lipid-associated Lck protein by myristoylation at p56. Lck activates the PLC-γ that breaks PIP3 into IP3 and DAG. IP3 triggers the opening of calcium channels in the ER and increases the concentration of Ca^2+^ in the cytosol. Increased Ca^2+^ activates the NFAT signaling. The virally encoded Vpr can also trigger the NFAT signaling through Ca^2+^ efflux and interferes with cGSK3β kinase for NFAT export (not shown). On the other hand, DAG via PKC activates the MAPK pathway. The NFAT and MAPK then promote the transcription of genes required for cytokine production and T-cell proliferation and activation. **(D)** The gp120-CD4 and CXCR4/CCR5 interaction upregulates the apoptotic receptor and ligand, Fas/FasL expression, which in turn activates the caspase 8 and caspase 3 for apoptosis of the infected cell via FADD/TRADD. **(E)** Additionally, the released viral Tat and Nef in the cytoplasm can also upregulate the Fas expression in the plasma membrane and can directly act on the caspase 8, promoting apoptosis.

During HIV-1 transmission, the virus utilizes the envelope glycoprotein and the chemokine co-receptors CXCR4 or CCR5, depending on the viral tropism, to gain an entry into the CD4^+^ T cells. The envelope glycoprotein gp120 establishes contact with the surface-expressed CD4, leading to conformational changes (Berger et al., 1999) that subsequently facilitate binding to co-receptors, a critical event for initiating a fusion apparatus (Figure 1, step 1). Binding of the co-receptor later results in conformational changes that enable the gp41 subunit to insert its hydrophobic fusion peptide into the host lipid membrane to drive the fusion process (Figure 1, step 2) (Doms and Moore, 2000; Waheed and Freed, 2009). The molecular mechanism of HIV-1 entry and viral membrane fusion are reviewed extensively elsewhere (Harrison, 2008, 2015; Kielian, 2014; Chen, 2019). The virus interplays with a myriad of host plasma membrane proteins. The host factors P-selectin glycoprotein ligand-1 (PSGL-1) and CD43 modulate HIV attachment to the plasma membrane by being incorporated into virions (Fu et al., 2020). HIV-1 encoded Vpu along with co-clustered Gag at the membrane downregulates PSGL-1 to exclude it from the virions to ensure efficient attachment to the target cell membrane. Interferon (IFN)-induced transmembrane proteins (IFITMs) constitute another IFN-inducible gene that has also been shown to interfere with the entry of HIV-1 by modulating fusion with the host membrane (Compton et al., 2014; Zhao et al., 2019). Retroviral envelope glycoproteins have the ability to alter the sensitivity of the virus from restriction by host factors that target early steps of the infection cycle like IFITMs and SERINC5 (Foster et al., 2016; Beitari et al., 2017; Firrito et al., 2018). The binding of HIV-1 to its receptor and co-receptors alone has shown to induce and alter a plethora of signaling pathways (Figure 2). For instance, pattern recognition receptor like NLRP3 inhibits F-actin remodeling and regulates the susceptibility to HIV-1 infection. Once the virus binds to its receptors, P2Y2 signaling is activated to mediate the degradation of NLRP3. In the absence of NLRP3, protein tyrosine kinase, PYK2, undergoes phosphorylation and activation, leading to a cytoskeletal rearrangement favorable for viral entry (Figure 2A; Paoletti et al., 2019). Moreover, the interaction of viral protein Nef and host-derived p21-activated kinase2 (PAK2) was found to play a role in activating NFAT and NF-κB transcription factors required for T-cell activation (Figure 2B) (Fenard et al., 2005). On the other hand, the binding of HIV-1 to its receptor and co-receptors myristoylates Lck at p56 and activates the PLC-γ (Figure 2C). This facilitates the breakdown of PIP3 into DAG and IP3. The DAG activates the MAP kinase pathway, whereas IP3 triggers the opening of Ca^2+^ channels in the ER. In addition, the virally encoded Vpr induces Ca^2+^ influx and promotes the nuclear import of NFAT. The NFAT and ERK activated by MAPK signaling then promote the transcription of genes for cytokine production and T-cell proliferation and activation (Höhne et al., 2016). Besides relaying cell signaling and host immune evasion, multiple reports emphasize the nature of HIV-1 that induces apoptosis by increasing the expression of membrane-bound Fas in T-cells and FasL in monocytes, macrophages, and NK cells during infection (Figure 2D) (Kottilil et al., 2007; Li et al., 2009). It was shown *in vitro* that these enhanced expression levels led to faster apoptosis via caspase 8 than the uninfected cells (Badley et al., 1996). Further, the virally derived Tat and Nef in the host cytosol increase the FasL level in the plasma membrane and directly activate caspase 3 and caspase 8 to promote apoptosis (Figure 2E) (Bartz and Emerman, 1999; Jacob et al., 2017). Altogether, just the binding and fusion of the virus with the host cell triggers a wider variety of pathways to trick the cell into creating a facile environment for HIV-1 replication.

## Cytoplasm: The Site of Commencement of Uncoating and Reverse Transcription

Successful binding and fusion with the plasma membrane result in the release of viral content into the cytoplasm of the host cell. In the cytoplasm, critical events of HIV-1 replication occurs, such as core delivery, reverse transcription, and translation (Figure 1, steps 3–4 and step 8). In this section, we attempt to give an insight into how HIV-1 adopts mechanisms to use or deceive the function of host cellular factors in core delivery and reverse transcription initiation.

### Initiation of Uncoating

Prevailing models suggested that for completing the reverse transcription of viral RNA, partial disassembly of CA protein is indispensable and that the uncoating event precedes the reverse transcription, though, until recently, the precise mechanism, timing, and location of uncoating remained contentious (Arhel, 2010; Ambrose and Aiken, 2014). Post-entry, in the cytoplasm, the HIV-1 core engages the host cytoskeleton for the commencement of uncoating and cytoplasm-nuclear trafficking (McDonald et al., 2002; Lukic et al., 2014; Delaney et al., 2017). In a yeast two-hybrid screening, the Arhel lab found two microtubule-associated proteins MAP1A and MAP1S, to bind to the CA of HIV-1 and to tether the virus to the microtubule network *en route* to the nucleus (Fernandez et al., 2015). Later, the same group identified that cellular β-karyopherin Transportin-1 (TRN-1) binding to the CA is necessary and sufficient for uncoating and efficient nuclear import (Fernandez et al., 2019). In addition, the host kinesin-1 adaptor protein, FEZ1, and dynein adapter protein, BICD2, interact with the CA and promote the uncoating of the core by pulling in opposite directions, as in “tug-of-war” (Lukic et al., 2014; Campbell and Hope, 2015; Malikov et al., 2015; Dharan et al., 2017; Carnes et al., 2018). Besides, two other cellular factors, Dia1 and Dia2, known to stabilize microtubules, interact with the CA and promote uncoating and DNA synthesis (Delaney et al., 2017). The completion of uncoating as a nuclear phenomenon will be discussed in detail with newer insights in later sections.

Numerous cellular factors are known to restrict retroviral infection (Malim and Bieniasz, 2012; Colomer-Lluch et al., 2018), one of which is a tripartite motif protein, TRIM5α, known to interfere with the uncoating and reverse transcription by interacting with the viral CA (Stremlau et al., 2004, 2006). The TRIM5α, in non-human primates, was shown not to hamper HIV-1 infection; however, the replacement of the PRYSPRY domain of TRIM5α by cyclophilin A (CypA) binding domain in New World owl monkeys restrict the HIV-1 infection strongly (Sayah et al., 2004; Stremlau et al., 2005; Balakrishna and Kondapi, 2016; Colomer-Lluch et al., 2018). Contrastingly, a recent discovery using primary human blood cells suggested that the interaction between CypA and CA is necessary to evade the restriction by TRIM5α. The absence of this interaction, however, decreases the viral infectivity in human cells (Kim et al., 2019; Selyutina et al., 2020b). The CypA is a peptidylprolyl isomerase that catalyzes the *cis*/*trans*-isomerization of the peptide bond between Gly89 and Pro90 of the CA domain of Gag and is known to prevent premature uncoating (Luban et al., 1993; Bosco et al., 2002). Such tricks played by HIV-1 against host cellular factors in different models suggest HIV as one of the clever viruses to alter the host cellular factors for its benefit.

### Commencement of Reverse Transcription

Following partial uncoating, reverse transcription begins (Figure 1, step 4) in an intricately organized manner forming an RT complex (RTC) in the host cytoplasm and completes in the nucleus just before successful uncoating (Figure 1, step 6) (Fassati and Goff, 2001; Burdick et al., 2020; Selyutina et al., 2020a). The RTC consists of viral RNA, host-derived tRNA^*Lys3*^ primer, eukaryotic translational elongation factor 1A (eEF1A), synthesized DNA, several viral factors, and host factors (Isel et al., 1996; Fassati and Goff, 2001; Balakrishna and Kondapi, 2016). The tRNA^*Lys3*^ works as a primer by binding to the 5′ primer binding site (PBS) in the vRNA and initiates the reverse transcription process with the help of several cellular factors such as integrase interactor 1 (INI1 and hSNF5), survival motor neuron (SMN)-interacting protein 2 (Gemin2), histone deacetylase 1 (HDAC1), and sin3A-associated protein (SAP18) (Isel et al., 1996; Balakrishna and Kondapi, 2016). Recently, David Harrich’s group reported the interaction between positively charged host eEF1A and the surface-exposed acidic E300 residue in the thumb domain of RT to play an essential role in viral uncoating, reverse transcription, replication, and infectivity (Rawle et al., 2018; Li D. et al., 2019). They also showed that E300R mutation or oxazole-benzenesulfonamide treatment reduces the RT interaction with eEF1A and thus delays the uncoating and reduces the viral reverse transcription and replication (Rawle et al., 2018, 2019). Once the minus-strand DNA is synthesized at the 5′ end, it is transferred to the 3′ end of the genome based on LTR’s repeated (R) region complementarity, where the minus-strand DNA synthesis is completed. During this synthesis process, the RNaseH activity of RT cleaves the RNA molecules except at central PPT (cPPT). The cPPT serve as the template for the synthesis of a dsDNA fragment. Following second-strand transfer, the plus-strand DNA synthesis continues till the central termination sequence (CTS), displacing almost 100 nucleotides of previously made DNA, generating a central DNA flap. Thus, the final product of the reverse transcription process in HIV-1 generates a dsDNA molecule with a flap in the center (Arhel, 2010).

Like TRIM5α, APOBEC3G and SAMHD1 acts as post entry restriction factors against HIV-1. APOBEC3G is encapsidated into the budding virions and is present in the RTC, inducing G-to-A hypermutation and fragmented cDNA production in a deaminase-dependent pathway. Besides, a deep sequencing strategy further revealed the role of APOBEC3G in a sequence- and site-independent interference with cDNA synthesis by direct interaction with the RT. Concomitant defective viral protein synthesis thus inhibits HIV-1 replication and assembly strongly (Sheehy et al., 2002; Pollpeter et al., 2018). While APOBEC3G-induced changes result in dysfunctional proteins, SAMHD1 depletes the cytoplasmic dNTP pool to hinder the reverse transcription process (Hrecka et al., 2011). To counteract these restriction factors, HIV1/2 encode accessory proteins like Vif and Vpx, which degrades APOBEC3G and SAMHD1, respectively, by employing Cullin E3 ubiquitin ligase complex (Sheehy et al., 2002; Hrecka et al., 2011). Further details on how HIV acts against other such restriction factors are described in the reviews of Malim and Bieniasz (2012) and Colomer-Lluch et al. (2018). Although the HIV-1 RNA is encapsidated within a core, several innate immune sensors are known to be activated upon capsid disruption. For instance, a member of the PYHIN family, IFI16 detects and binds to the incomplete HIV-1 cDNA and triggers the STING-TBK1-IRF3 signaling axis to promote the transcription of antiviral genes in myeloid cells. However, considering recent understanding of the completion of reverse transcription within the nucleus, the IFI16 sensing mechanism may have to be reconsidered. IFI16, in addition, triggers IL-1β production and promotes CD4^+^ T cell death via ASC and caspase-1 in lymphoid cells (Jakobsen et al., 2013). Another cytosolic DNA sensor, cyclic GMP-AMP synthase (cGAS), is widely known for its antiviral immunity in the context of HIV-1 infection. cGAS preferentially detects abruptly formed HIV-1 reverse-transcribed DNAs in monocyte-derived dendritic cells (DCs) via polyglutamine binding protein-1 (PQBP1) and triggers the IFN response against HIV-1 through the STING-TBK1-IRF3 signaling pathway. However, HIV-1 suppresses the cGAS-STING activation by exploiting the NOD-like receptors family, NLRC3, an ATPase that promotes the sequestration and attenuation of STING activation and thus inhibits the transcription of IFN (Barouch et al., 2016). Moreover, recently, it was found that even though SAMHD1 acts as a restriction factor, it promotes the degradation of nascent incomplete HIV-1 DNA, and prevents the activation of cGAS-STING-mediated IFN production. Similarly, a ubiquitously expressed three prime repair exonuclease 1 (TREX-1) acts on incomplete reverse transcription products and prevents the cGAS-STING activation (Kumar et al., 2018; Chen et al., 2019). Further, the integrity and stability of CA along with the host cleavage and polyadenylation specificity factor 6 (CPSF6) and cyclophilins physically protect the viral reverse transcripts in the cytoplasm from cGAS and thus inhibits the production of type I IFNs (Rasaiyaah et al., 2013; Sumner et al., 2020). To understand the various stratagem employed by HIV-1 against cellular immunity, readers are encouraged to follow the recent review by Yin et al. (2020).

## Nuclear Interactions

### Cytoplasm to Nuclear Import and the Process of Uncoating and Reverse Transcription Completion

To integrate viral genomic DNA into the host chromosome, prior CA uncoating becomes indispensable. The exact location of uncoating and the precise timing of reverse transcription are incompletely understood. Based on earlier findings, different uncoating models were proposed and are explicated in the reviews of Arhel (2010), Ambrose and Aiken (2014), and Campbell and Hope (2015). One of the prevailing models of uncoating suggests that the viral core is trafficked to the cytoplasmic side of the nuclear envelope by the host microtubules and host factors such as FEZ1 and BICD2, where the uncoating occurs at the nuclear pore complex (NPC). The capsid is disassembled after uncoating, leaving the viral genetic material complexed with the host and viral proteins. This nucleoprotein complex is known as PIC and is protected from nuclease degradation and innate sensing in the host cell (Khiytani and Dimmock, 2002; Arhel, 2010; Malikov et al., 2015; Dharan et al., 2017). The uncoating process and docking at NPC are in agreement with earlier work from the Melikyan laboratory, where authors showed the importance of CA in these events. They also reported the proteasomal degradation of HIV-1 complexes if uncoating happens in the cytoplasm (Francis and Melikyan, 2018). The uncoating at NPC and trafficking to the nucleus are mediated by the interaction of viral CA with nucleoporin, NUP-153, and the coordinated facilitation between NUP-358 and kinesin-1 family, KIF5B (Brass et al., 2008; König et al., 2008; Dharan et al., 2016; Burdick et al., 2017). Besides, TRN-1, a β-karyopherin, was identified to bind to the CA, promoting uncoating and subsequent nuclear import (Fernandez et al., 2019). Similarly, another TRN, TNPO3 (also known as TRN-SR2), now known to play a role during integration, also associates with the CA and promotes uncoating and nuclear trafficking by regulating the localization of cellular protein CPSF6 (Brass et al., 2008; König et al., 2008; Price et al., 2012; De Iaco et al., 2013; Chin et al., 2015). For further details into the older understanding of uncoated core trafficking into the nucleus, the readers are encouraged to refer to Ambrose and Aiken (2014), Campbell and Hope (2015), and Novikova et al. (2019).

However, the latest findings of Burdick et al. and Selyutina et al. revealed that the intact viral core (or nearly intact) is trafficked into the nucleus with the assistance from the CPSF6 (Figure 1, step 5) and uncoats < 1.5 h prior to integration at the proximity of 1.5 μm from the sites of integration (Figure 1, step 6). Their findings also stress the fact that the process of reverse transcription completes within the nucleus at SC35 nuclear speckles before the completion of uncoating (Lahaye et al., 2018; Burdick et al., 2020; Selyutina et al., 2020a). Preceding this study, using primary human macrophages, Bejarano et al. showed that CPSF6 is excluded from the cytoplasmic RTC/PIC; however, they are present in the nuclear replication complexes. Moreover, the reduction in CPSF6 leads to the accumulation of HIV-1 particles at the nuclear envelope. They also established that CPSF6 directly interacts with the CA and induces the nuclear import of the viral complex (Bejarano et al., 2019). This interaction also decides the integration site of the proviral DNA in the host euchromatin. The disruption of CA–CPSF6 interaction led to integrating viral DNA in the heterochromatin region of the host chromosome (Burdick et al., 2020). Further, independently, other researchers have claims supporting the observations that nuclear import precedes the reverse transcription and uncoating process (Dharan et al., 2020; Selyutina et al., 2020a). Collectively, all these new findings change our understanding of HIV-1 infection and post-entry events.

Similar to every other step, the host thwarts the HIV-1 life cycle at the nucleus as well. Myxovirus resistance 2 (MX2/MXB), an IFN-induced post-entry inhibitor of HIV-1, was found to act as an antiviral host factor by blocking the nuclear import of viral cDNAs. This MXB sensitivity was found to be dependent on the conformation of HIV-1 CA, but how exactly HIV-1 overcomes this hurdle is yet to be elucidated in detail (Goujon et al., 2013; Kane et al., 2013; Dicks et al., 2018; Miles et al., 2020). In addition, the TRIM5 interacts with the CA and activates protein kinase enzyme TAK1, which in turn activates the activator protein 1 (AP-1) and NF-κB innate immune signaling pathway (Sultana et al., 2019; Yin et al., 2020). Further, Lahaye et al. (2018) found the binding of host NONO with the HIV-1 and HIV-2 nuclear monomeric CA, HIV-1 DNA, and cGAS to trigger the production of IFN inside the nucleus. These findings support the previously mentioned nuclear model of uncoating and reverse transcription (Lahaye et al., 2018). Crossing these obstacles to gain an entry into the nucleus and successful uncoating, the HIV-1 integrates its genome into the host chromosome to complete the process of transcription, one of the major events in the HIV-1 life cycle. Thus, in the following subsections, we attempt to review the current knowledge about how integration, transcription, latency, and latency reactivation occurs inside the nucleus.

### Integration of Viral DNA Into the Host Chromosome

Once inside the nucleus, the HIV-1 modulates the nuclear environment for viral cDNA integration into the host chromosome as a provirus (Figure 1, step 7), specifically at the AT-rich euchromatin region and other active transcriptional units (Craigie and Bushman, 2012; Balakrishna and Kondapi, 2016; Ciuffi, 2016). The viral protein IN mediates the process of integration, and the IN is destabilized by cellular E3 RING ligase TRIM33, preventing the formation of provirus (Ali et al., 2019). In addition, the host polypyrimidine tract binding protein and associated splicing factor (PSF) binds to the HIV-1 IN-cDNA complex and destabilizes the complex, suppressing the integration event (Yadav et al., 2019). On the other hand, the host lens epithelium-derived growth factor (LEDGF/p75) binds to the IN and directs the integration of viral cDNA at transcriptionally active sites by interacting simultaneously with the host chromatin (Llano et al., 2006; Ciuffi, 2016). The component of SWI/SNF chromatin remodeler, INI1, then interacts with the IN domain of Gag-Pol protein and promotes the DNA joining activity of IN (Turelli et al., 2001; Yung et al., 2004). In LEDGF/p75 depleted cells, HIV-1 utilizes hepatoma-derived growth factor-related protein 2 (HRP-2) for successful integration; however, this process’s efficiency is significantly less (Schrijvers et al., 2012a,b). In addition to LEDGF/p75, HIV-1 also influences other host factors such as high-mobility group protein A1 (HMGA1), HMG I(Y), barrier-to-auto-integration factor (BAF), SUV39H1, EED, and HP1γ for the integration process (Farnet and Bushman, 1997; Lin and Engelman, 2003; Du Chéné et al., 2007). Further, as described above, fresh observations regarding the role of CPSF6 in integration also determine the fate of integrated proviral DNA (Bejarano et al., 2019; Burdick et al., 2020). It has been hypothesized that CA–CPSF6 interaction facilitates the HIV-1 to the gene-rich regions, whereas IN-LEDFG/p75 explains the preference for integration in the gene bodies. Of note, it is not always that PIC in the nucleus is favored for the process of integration. Sometimes, the PIC dissociates, leaving the two ends of the viral cDNA to get ligated by the host non-homologous DNA end-joining mechanism (NHEJ), forming a 2-long LTR circles. These 2-LTR circles represent the dead ends for the virus and are overcome by host LEDGF/p75 (Farnet and Haseltine, 1991; Li et al., 2001). The molecular mechanisms of integration are reviewed in detail elsewhere (Kvaratskhelia et al., 2014; Ciuffi, 2016; Poletti and Mavilio, 2018). Taking this into consideration, like in the other steps of the viral life cycle, the host tries to prevent provirus formation. However, the virus influences the host factors, especially chromatin-binding proteins, to integrate its genome into the host chromosome successfully. Downstream to integration, another crucial event in the viral life cycle is described below, where the provirus is transcribed into RNAs for making several progenies of its own.

### Transcription and Latency

Following successful integration, the virus has two possibilities: it either goes for active transcription and production of virions, or undergoes latency and remains silent if inefficient transcription occurs. The viral transcription (Figure 1, step 8) is a crucial step that recapitulates the host transcription in many aspects, especially by manipulating most of the host transcriptional machinery. The process commences by recruiting host RNA polymerase (pol) II at 5′-LTR and several other transcriptional regulators such as NF-κB, NFAT, AP-1, and SP-1 at their respective binding sites upstream to the LTR promoter. These regulators work synergistically to ensure the viral gene expression while minimizing the host’s antiviral gene activity (reviewed in Ruelas and Greene, 2013; Van Lint et al., 2013; Röling et al., 2016). Blocking any of the ways by which transcription is favored, such as by adding repressive chromatin marks, epigenetic silencing, limiting positive transcription factors, or excessively supplying negative transcriptional regulators, leads to the inhibition of viral DNA transcription resulting in latency. The post-integrated latent virus has since then been a bottleneck for using antiretroviral therapies (ARTs) for achieving a complete cure. This priority research area, the mechanism of latency, and approaches to treat the latently infected cells are well rationalized in Coiras et al. (2009), Liu et al. (2014), Cary et al. (2016), Mbonye and Karn (2017), Lindqvist et al. (2020), and Shukla et al. (2020). The latency at any later time point does relive and can reactivate the integrated HIV-1 for transmission.

In both fresh and reactivated transcription processes, the pol II at 5′-LTR transcribes the stem loop of transactivating response (TAR) element and halts due to secondary structures, generating abortive transcripts. This halting is vanquished by recruiting positive transcription elongation factor b (P-TEFb) by Tat at the TAR element. The P-TEFb is a heterodimer of cyclin-dependent kinase 9 (CDK9) and cyclin T1 (CycT1) that phosphorylates the c-terminal domain (CTD) of RNA pol II and thus favors the elongation process producing full-length HIV-1 transcripts (Jones and Peterlin, 1994; Jones, 1997; Garber et al., 1998; Bieniasz et al., 1999; Zhou et al., 2000). Since P-TEFb is required for both viral and cellular gene expression, its tight control in the cell is indispensable. In most of the cells, P-TEFb is in an inactive state and is sequestered in a kinase-inactive complex that contains hexamethylene bis-acetamide inducible 1 (HEXIM1), and this P-TEFb–HEXIM1 interaction is mediated by 7SK small nuclear RNA as a molecular scaffold. Besides, the kinase-inactivated complex also contains Lupus antigen (La)-related protein 7 (LARP7), a methyl phosphate capping enzyme called MePCE, AF9, AFF1, AFF4, ENL, ELL1, and ELL2. Together, this entire complex is known as super elongation complex (SEC) (Nguyen et al., 2001; Yik et al., 2004; He et al., 2010; Liu et al., 2014). In an infected cell, the P-TEFb dissociates from the SEC and forms an association with the bromodomain-containing protein 4 (Brd4). Brd4 then facilitates the recruitment of P-TEFb at the promoter site for Tat-independent transcription stimulation (Yang et al., 2005). However, it is compelling to note that in the presence of Tat, Brd4 plays a negative role in the transcriptional process by competing with the Tat (Yang et al., 2005; Bisgrove et al., 2007). A decade ago, work led by the D’Orso group revolutionized the understanding of how and when Tat and P-TEFb are recruited to the HIV promoter. Their studies showed that even before TAR element formation, Tat, in association with P-TEFb, is mobilized to the 5′-LTR promoter in a specificity protein 1 (SP1)-dependent manner facilitating the transcription process (D’Orso and Frankel, 2010; D’Orso et al., 2012; McNamara et al., 2013).

Initially, it was reported that TRIM22 has a broad antiviral activity, inhibits SP1, and thus represses the transcription (Turrini et al., 2015). More recently, it was revealed that IFI16 sequesters the SP1 transcription factor concurrently, inhibiting the viral gene expression (Hotter et al., 2019; Bosso et al., 2020). Besides, a short isoform of Per-1 was identified to suppress the transcription process in resting CD4^+^ T cells. However, this suppression is overcome by the activity of Tat (Zhao et al., 2018). Taura et al. (2019), in a recent finding, showed an unexpected role of APOBEC3A in inducing latency. The APOBEC3A interacts with the proviral 5′-LTR and adds repressive histone marks by recruiting HP1 and KAP1. In addition, a member of the heterogeneous nuclear ribonucleoproteins (hnRNPs) family, X-linked RNA-binding motif protein (RBMX), was found to bind to the LTR downstream region and to block the recruitment of RNA pol II at the promoter by maintaining repressive trimethylation of histone H3 lysine 9 (H3K9me3) (Ma et al., 2020). Further, a recent CRISPR-based knockout screen by Rathore et al. (2020) revealed the role of several host deubiquitinases such as UCH37, USP14, OTULIN, and USP5 in HIV-1 latency. In the lymph node, where oxygen availability is less, Zhuang et al. (2020) showed that hypoxia-inducible factor 2α (HIF2α) binds to LTR, suppresses the transcription, and promotes latency. These studies further await independent confirmations on the factors identified to regulate the latency. On the contrary, several other findings suggested the novel mechanism of reactivation of latent HIV-1. For instance, ELL2 being the part of SEC, however, stimulates the transcriptional elongation, but the freshly synthesized ELL2 is prone to degradation by Siah1. This inhibitory activity of Siah1 is antagonized by host cell factor 1 and 2 (HCF1 and HCF2), thus favoring the transcriptional activation (Wu et al., 2020). Additionally, the same group also suggested that the levels of ELL2 and ELL2-SEC can be elevated by downregulating/inhibiting the proteasomes favoring Tat transactivation (Li Z. et al., 2019). Interestingly, another finding suggests that YY1 is known to inhibit HIV-1 expression and to promote latent infection, which, when over-expressed, leads to transcriptional upregulation with the synergistic effect of viral Tat protein (Yu et al., 2020). Another viral accessory protein, Vpr, was found to reactivate HIV-1 by targeting the chromatin-modifying enzyme CTIP2 (Forouzanfar et al., 2019). Taken together, the consequent HIV-1 transcription being either active or silenced depends mostly on host cellular factors, epigenetic factors, and viral factors in addition to the chromatin status at the integration site.

### Splicing and Export of Viral Transcripts to Cytoplasm

Upon completion of transcription, a full-length mRNA transcript (∼9.2 kb) is produced containing eight open reading frames (ORFs). The transcript then undergoes alternative splicing to form Rev, Tat, and Nef mRNA (∼1.8 kb) by a mechanism similar to that of the host (Chen and Manley, 2009; Kutluay et al., 2014; Sertznig et al., 2018). The Rev mRNA is transported out of the nucleus through the NPC and is translated into Rev proteins in the cytoplasm (Köhler and Hurt, 2007). The Rev protein-containing NLS is imported back to the nucleus by binding to the nuclear import receptor, importin β (Henderson and Percipalle, 1997).

In the late phase of infection, when the concentration of Rev protein in the nucleus is above a certain threshold, it binds to the Rev response element (RRE) in the second intron of unspliced and incompletely spliced transcripts (Pollard and Malim, 1998). The Rev also contains a nucleus export signal (NES) through which it binds to the karyopherin family member exportin 1 [also known as chromosome maintenance region 1 (CRM1)] and transport the transcripts from the nucleus to the cytoplasm (Fischer et al., 1995; Arnold et al., 2006). Of note, Rev multimerizes and masks the NES, which thus can be retained in the nucleus (Behrens et al., 2017). It was later discovered that RRE–Rev interaction also recruits hypermethylation enzyme PIMT, which modifies the 7-methylguanosine (m7G) cap of mRNA to a trimethylguanosine (TMG) cap. The acquisition of TMG caps allows the HIV-1 RNA to get recognized by CRM1 and targets for CRM1-dependent nuclear export (Yedavalli and Jeang, 2010). In addition to several host factors (such as DDX1, DDX3, DDX5, DDX21, Matrin-3, CBP80, Sam68, and MOV10) found to interact with Rev–RRE, Wang et al. (2019) found two proteins, ANP32A and ANP32B, which directly interact with RRE–Rev–CRM1 and facilitate the viral RNA nuclear export. The HIV-1 Nef-associated factor 1 (Naf-1, a cellular protein) was also found to interact with CRM-1 and promote the export of HIV-1 gag mRNA (Ren et al., 2016). Exported HIV transcripts then undergo translation and encode viral structural proteins (Gag and Pol from unspliced RNA) and accessory proteins from singly spliced transcripts (Env, Vif, and Vpr). These viral proteins are then trafficked via different cellular compartments to the virion assembly site at the plasma membrane.

## Cytoplasm: The Site of Viral Protein Translation and Interaction With Other Organelles

### Translation

Besides using the cytoplasmic environment for initiating reverse transcription and traversing the core toward the nucleus, HIV-1 also utilizes cytoplasm for viral translation and assembly (Figure 1, steps 9–10), after the successful production of viral RNA and their export to the cytoplasm. Prior to translation initiation, HIV-1 encounters several hurdles elicited by the host cellular environment as a result of the innate sensing of virion components. To limit viral production, the host induces the production of IFN-stimulated gene products. Following cellular stress, protein activator of PKR (PACT) activates an IFN-activated protein kinase (PKR) and mounts antiviral immunity (Burugu et al., 2014; Guerrero et al., 2015). However, a few years back, Chukwurah et al. (2017) showed that HIV employs a strategy to subdue this antiviral response by interacting viral Tat protein, host ADAR1, and PACT, inhibiting the PKR activation and thus enhancing own protein synthesis. Earlier, we have discussed the role of IFITMs in the inhibition of viral entry, but Lee et al. recently unveiled the translational inhibition role for IFITMs. The IFITM excludes the viral mRNA from incorporation into the polysomes and thus inhibits the protein synthesis. Furthermore, as a countermeasure, Lee et al. (2018) found that HIV Nef overcomes this inhibitory effect by IFITM by a mechanism not known yet.

For translation initiation and protein production, HIV-1 misappropriates the eukaryotic translational machinery by recruiting 40S ribosomes to its 5′ UTR region of RNA, which is capped (secondary structure) like host RNAs. This region is known as the TAR element required for translation initiation. However, similar to eukaryotic translation, the presence of highly stable RNA structures in the viral TAR RNA region has raised several questions about the recruitment of the 43S pre-initiation complex (PIC). In eukaryotes, the 43S PIC containing 40S ribosome, initiator tRNA, eIF1, eIF1A, eIF2-GTP, and eIF3, is recruited to the 5′ Cap by eIF4F multi-subunit complex and facilitates the scanning of mRNA for initiator codon from 5′ to 3′ direction. Interestingly, HIV-1 masters itself by recruiting a cellular RNA helicase DDX3 (DEAD/H box family) and facilitates PIC assembly in an ATP-dependent manner (Ricci et al., 2008; Guerrero et al., 2015). HIV-1 also recruits host TAR RNA-binding protein (TRBP—known to be involved in RNA silencing) at TAR elements and resolve the secondary structure for translation initiation. This year, Komori et al. (2020) showed that TRBP interacts with the DICER and mediates TAR miRNA degradation, reliving the hurdle. Apart from canonical translation initiation, eukaryotes and many viruses, including HIV-1, employ a cap-independent translation mechanism. In this process, the 43S initiation complex is recruited at the internal ribosome entry site (IRES) containing mRNA stem loops to initiate the translation in a cap-independent manner. The HIV-1 virus, in the first 24–48 h of viral replication, uses cap-dependent translation process, whereas after 48 h, it opts for the IRES-dependent translation process to produce viral particles (Amorim et al., 2014; Ohlmann et al., 2014). For further understanding of the molecular mechanism of translation initiation, elongation, and completion, readers are encouraged to follow the articles by Ohlmann et al. (2014) and Guerrero et al. (2015).

Altogether, these studies show that apart from using host cellular factors and plasma membrane for viral entry and budding, the virus tricks the host machinery and makes the host environment favorable for viral replication. Post translation, the viral proteins are targeted to several cellular organelles for protein modifications. These modified proteins are then transported to the plasma membrane for assembly and virion production. In the upcoming section, we attempt to describe various tricks played by HIV-1 within these organelles for its benefit.

### The Interactions With the Endoplasmic Reticulum, Mitochondria, and Peroxisome

Preparatory to the assembly of virions, HIV-1 proteins are synthesized on the endoplasmic reticulum (ER) and are targeted to various cellular compartments for protein modification, maturation, and alteration of immune pathways. For instance, the HIV-1 uses the host ribosome machinery bound to the ER to produce gp160, an Env polyprotein precursor. The gp160 is then glycosylated in the ER, concomitant with translation, and multimerizes for trafficking to the TGN. In Golgi, the precursor proteins undergo oligosaccharide modification and are processed to yield transmembrane glycoprotein, gp41, and surface glycoprotein, gp120 (Checkley et al., 2011). To prevent premature interaction of gp120 with newly synthesized CD4 on ER, HIV-1 employs Vpu to manipulate the β-TrCP/proteasome-mediated degradation pathway to downregulate CD4 (Margottin et al., 1998; Magadán et al., 2010). Of note, CD4 receptors in the cell surface are downregulated by viral Nef protein by hijacking adaptor protein complex 2 (AP2)- and clathrin-dependent endocytosis (Kwon et al., 2020).

It is interesting to note that, although HIV-1 seizes ER for protein synthesis, glycosylation, and CD4 downregulation via ERAD machinery, the ERAD acts as a double-edged sword that traps gp160 at its birthplace. Besides, in the ER, the guanylate-binding protein, GBP2/5, decreases the activity of furin convertase required for conversion of precursor gp160 into mature gp41 and gp120 (Braun et al., 2019). An ER protein, known as ERManI, modulates the glycosylation of Env protein vis-à-vis regulating TSPO, a mitochondrial translocator protein that alters the folding process and diminishes Env expression by ERAD (Zhou et al., 2014, 2015). This suggests that mitochondrial involvement in regulating the Env protein folding process. Currently, we do not know how exactly HIV-1 responds to this, but recent findings by Zhang et al. (2016) showed that HIV-1 accessory protein Vpr augments proper Env folding in the ER that, in turn, shields Env from lysosomal degradation in the ERAD pathway. Another study showed that HIV-1 hijacks PERK, ATF6, and IRE1 ER stress sensors and modulates their activity to increase BiP expression and subsequent increased protein folding capacity of ER (Borsa et al., 2015).

**FIGURE 3:**
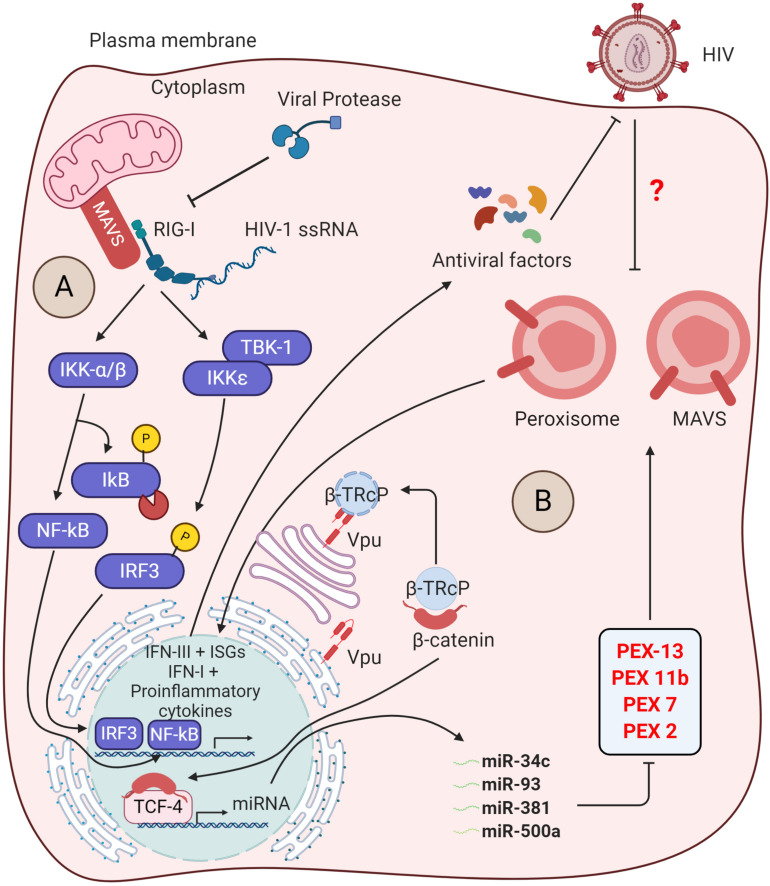
Insights into the counteraction of host defense by viral protease and Vpu. **(A)** Inhibition of antiviral signaling by viral protease: The host RIG-I senses viral ssRNA to promote the antiviral signaling through MAVS and activates the IκB kinases (IKKs). The IKKε phosphorylates IRF3, which then translocates into the nucleus to trigger the IFN-I production. On the other hand, IKK-α/β phosphorylates and degrades IκB, reliving NF-κB to go into the nucleus for transcription of proinflammatory cytokine genes. The viral protease (PR) promotes the degradtaion of RIG-I in the cytosol. **(B)** Suppression of peroxisome biogenesis by Vpu: in the absence of HIV-1 infection, the adapter protein βTrCP binds to β-catenin and promotes its degradation via ubiquitin-mediated proteasomal pathway. Upon HIV-1 infection, the Vpu stabilizes the β-catenin by sequestering βTrCP. Subsequently, β-catenin enters into the nucleus and activates the transcription factor TCF-4, which is required to drive the expression of indicated microRNAs. These microRNAs were found to regulate the expression of peroxisome biogenesis factors for peroxisome synthesis. However, the peroxisomal MAVS triggers the rapid induction of type III IFNs downstream ISGs that acts as antiviral factors. The direct/indirect counteraction of peroxisomal MAVS signaling by HIV-1 remains to be elucidated.

In addition to this, HIV-1 was also found to manipulate the ERAD pathway and other innate immune triggering pathways to antagonize the immune responses as described in Byun et al. (2014) and Yin et al. (2020). In myeloid cells, the adaptor protein mitochondrial antiviral-signaling protein (MAVS) transduces signals from cytosolic RIG-I upon sensing viral RNAs that induce IRF3 and IκB activation. This leads to the activation of mitochondrial MAVS-mediated innate immunity (Figure 3A). The MAVS triggers the type I IFN signaling by another viral RNA sensor, DDX3, which interacts with the abortive HIV-1 RNA upon infection. However, HIV-1 utilizes the viral protease to diminish RIG-I from the cytosol, thus subverting RIG-I-MAVS initial signaling cascades. Additionally, HIV-1 sensing by host DC-SIGN activates a mitotic kinase PLK1 that lessens the downstream cascade signaling of MAVS, thereby escaping innate immune activation during HIV-1 infection. PLK1-mediated viral subversion strategy prevents DDX3-MAVS signaling, thereby promoting HIV-1 replication during infection (Gringhuis et al., 2016).

Intriguingly, MAVS signaling not only is limited to the mitochondrial membrane but also indulges in peroxisome membranes. Upon HIV-1 RNA sensing, peroxisomal MAVS triggers the rapid induction of type III IFNs and ISGs that acts as antiviral factors (Hopfensperger and Sautera, 2020). To antagonize the peroxisomal MAVS-mediated immunity, HIV-1 directly modulates the biogenesis of peroxisome factors. The viral accessory protein Vpu sequesters β-TRcP and stabilizes the β-catenin, required for activation of TCF4 TF to transcribe miRNAs (miR-34c-3p, miR-93-3p, miR-500a-5p, and miR-381-3p). These miRNAs are found to regulate the expression of factors required for peroxisome biogenesis and, thus, expropriates the peroxisome function (Figure 3B) (Xu et al., 2017, 2020; Hopfensperger and Sautera, 2020). However, whether the suppression of peroxisome biogenesis by Vpu inhibits the peroxisomal MAVS signaling and activation of IFN-stimulated genes (ISGs) and type III IFN is yet to be determined. Being an enveloped virus, HIV-1 may rewire peroxisome features to enhance lipid synthesis for new viral assembly.

Apart from antagonizing MAVS-dependent signaling, the HIV-1 employs accessory protein Vpr, Tat, and envelope glycoprotein gp120 to induce host cell death by altering the mitochondrial dynamics, membrane potential, and oxygen consumption. The Vpr reduces the expression of mitofusin 2 (Mfn2) post-transcriptionally, thus weakening and increasing the permeability of mitochondrial outer membrane (MOM). This leads to increased mitochondrial deformation and a reduction in mitochondrial membrane potential (MMP). Vpr also decreases the cytoplasmic level of fission protein, dynamin-related protein 1 (DRP1), and increases the bulging in membranes of mitochondria associated with mitochondria, MAMs. This suggests that Vpr-mediated cellular damage is modulated by DRP1 and MFN2 on an alternative protein transport pathway from the ER to mitochondria via mitochondria-associated membranes (MAMs) (Huang et al., 2012). Like Vpr, Tat and gp120 were found to alter the mtDNA content, mitochondrial dynamics, function, distribution, and trafficking. Tat and gp120 were also shown to induce the expression of mitophagy signaling proteins (DNM1L, PRKN, and PINK1) and autophagosome-related proteins (MAP1LC3B-II and BECN1). However, the increase in Parkin/SQSTM suggests the blockade in mitophagy flux and thus the accumulation of mitophagosomes in neurons (Avdoshina et al., 2016; Rozzi et al., 2018; Teodorof-Diedrich and Spector, 2018; Thangaraj et al., 2018). Additionally, HIV-1 promotes mitochondrial dysfunction for infection-mediated apoptosis by downregulation of mitochondrial complex I subunit NDUFA6 and the complex I enzyme activity (Ladha et al., 2005). HIV-1 protease has also been shown to play a role in apoptosis by localizing to the mitochondria and decreasing the MMP, following which it activates caspase 9, PARP cleavage, and DNA fragmentation (Rumlová et al., 2014). Furthermore, the HIV-1 gp120 induces the caspase-9/caspase-3-mediated programmed cell death by JNK, IRE1α, and AP-1 pathway by upregulating CHOP and BiP production (Shah et al., 2016). Altogether, emerging evidence suggests that HIV-1 exploits cytoplasm and nucleus, targets other subcellular compartments, and alters canonical cellular pathways for the completion of its life cycle.

## Plasma Membrane: The Site of Virion Assembly and Budding

### Assembly

In the later stages of the HIV-1 life cycle, post-translation and protein modification, the virus utilizes the inner leaflets of the host plasma membrane for assembly of HIV-1 (Figure 1, step 10). The gag protein of a virus consisting of MA, CA, and NC protein is essential for virion assembly. The Gag is translated from the viral RNA by programmed ribosome-1 frameshifting via two regimes established by Korniy et al. (2019). This frameshifting event is required for maintaining a constant Gag to Gag-Pol ratio for proper structural organization and infectivity of the virions. Besides, the cellular polyanion, inositol hexakisphosphate (IP6), interacts and enhances the assembly of Gag proteins into the immature viral particles (Dick et al., 2018; Mallery et al., 2019). During assembly, the viral RNA is recognized by the NC domain of uncleaved Gag protein via two zinc finger motifs and several basic amino acids and is selectively incorporated in the virions. Although HIV-1 RNA serves as a viral genome and template for translation, at a given time, a single RNA molecule carries out only one function (Bell et al., 2012; Kutluay et al., 2014; Chen et al., 2020). Recent studies indicate that viral RNA also interacts with the MA, leading to a reduction in the non-specific binding of Gag to the plasma membrane (Meng and Lever, 2013). Immediately after translation, Gag protein forms complexes with the two RNA granule proteins ABCE1 (ATP-binding cassette family of protein subfamily E1) and DDX6 (DEAD-box RNA helicase) present in the cytoplasm of infected cells. ABCE1 is a cellular ATPase and binds Gag independent of viral RNA, and its association with Gag protein during assembly indicated the energy-dependent polymerization of Gag monomers (Abrahamyan et al., 2008; Meng and Lever, 2013). The role of DDX6 during HIV-1 assembly still needs to be further studied. Another protein, Staufen1, is an RNA binding protein that indirectly binds to viral gag RNA and helps in gag oligomerization (Cochrane et al., 2006; Abrahamyan et al., 2008). Inhibition, as well as overexpression of Staufen1 protein, inhibits virus infectivity. Further, Staufen1, along with ABCE1 and DDX6, helps in Gag multimerization. Interestingly, these proteins only help during the assembly of HIV-1 but are not packaged in the budded virions (Abrahamyan et al., 2008; Meng and Lever, 2013; Lingappa et al., 2014).

### Swindling Cellular Factors During Virion Egress

Apart from entry into the target cell and harnessing the plasma membrane for assembly, HIV-1 also exploits the plasma membrane during budding from the producer cells (Figure 1, step 11). The budding process ensues the release of viral progeny from the infected cell, which will further help the virus disseminate the infection to new target cells. During egress, the PTAP motif in L-domain of HIV-1 gag p6 interacts with host tumor susceptibility gene 101 (TSG101), apoptosis-linked gene 2-interacting protein X (AIP1/ALIX), and endosomal sorting complexes required for transport (ESCRT) machinery, promoting the budding event (Garrus et al., 2001; Strack et al., 2003; Martin-Serrano and Neil, 2011). The budding requires all ESCRT-1 complex components, which consist of TSG101 VPS28, VPS37, and MVB12; and the latest member to this is the ubiquitin associated protein-1 (UBAP-1) (Ahmed et al., 2019). Recent studies revealed that mutation in NC leads to the delocalization of TSG101 but not ALIX1, suggesting that the distribution and interaction of TSG101 are Gag dependent (El Meshri et al., 2018). HIV-1 recruits the charged multivesicular body protein 4 (CHMP4) fission factor, an ESCRT III protein via ESCRT-1 at PTAP late domain. Besides, HIV-1 recruits two small subunits of ESCRT-III, CHMP2a, and CHMP2b. The recruitment of ESCRT-III is facilitated by the interaction of the C-terminal domain of CHMP4 with ALIX1 at the membrane, which further enables the formation of ESCRT-III filaments (Martin-Serrano and Neil, 2011). The ESCRT-III of ESCRT machinery acts as the key scissor to cut the filament, which then separates the nascent virion from the host plasma membrane. The vacuolar protein sorting associated protein 4 (VPS4) then continuously removes the ESCRT-III molecules from the excision site until membrane fission and virion release (Figure 1, step 12) is completed for another round of the budding event. For further insight into the event of budding, the readers are encouraged to have a look into the articles by Pincetic and Leis (2009), Martin-Serrano and Neil (2011), Weiss and Göttlinger (2011), and Lee et al. (2015). Recently, Popov et al. (2018) demonstrated that independent of the ESCRT-mediated budding process, the p6 region also recruits host PACSIN2, an actin cytoskeleton, and cellular membrane remodeler, via ubiquitin to promote cell-to-cell virion spreading, and this p6 domain ubiquitination was found to be facilitated by NEDD4 family ubiquitin ligase ITCH. Although being the predominant mode of transmission, the mechanism is yet to be understood in detail and thus opens up several questions in the biology of HIV-1 budding. At this stage, while the virions are ready to excise and leave the infected cells, this egress is challenged by the cellular protein tetherin (Neil et al., 2008; Van Damme et al., 2008). Tetherin is an IFN (IFN)-induced host protein, encoded by the BST-2 gene, known to sense the viral particles by transducing signals to activate proinflammatory signaling (Skasko et al., 2011; Galão et al., 2012). Tetherin cross-links the enveloped viruses during budding from the infected cell and thus inhibits the process. HIV-1 accessory protein Vpu counteracts this block at the plasma membrane by downregulating tetherin from the cell surface and promoting its degradation by recruitment of β-TrCP2 (Douglas et al., 2009).

Similar to Vpu, HIV-1 accessory protein, Nef has been extensively studied for its ability to alter cell surface protein composition. The primary function of Nef is known for trafficking a myriad of proteins from the cell surface to the trans-Golgi network or lysosome by hijacking the vesicular endocytic machinery. One such crucial function is the downregulation of the CD4 receptor by expropriating the endocytosis process, upon which the susceptibility of gp120 epitopes to host antibodies diminishes, thereby preventing antibody-dependent cellular cytotoxicity (ADCC) (Wyatt et al., 1995; Ferrari et al., 2011; Pham et al., 2014; Veillette et al., 2014; Kwon et al., 2020). Furthermore, by an uncharacterized mechanism, Nef was also shown to enhance virion’s infectivity by showing its effect in the HIV-1 producer cells rather than in the viral progeny itself (Chowers et al., 1994; Basmaciogullari and Pizzato, 2014). In 2015, host restriction factors SERINC3 and SERINC5 were identified. These multipass proteins dramatically inhibited Nef defective HIV-1 infectivity in target cells by being incorporated into the virus particle. In the presence of Nef, these cell-surface proteins are downregulated to late Rab-7 positive endosomal compartments and prevent the incorporation of these proteins into the budding virions. While infectivity defect is inherited during the egress from the producer-cell plasma membrane, the effect on virus inhibition is seen in the target cells (Rosa et al., 2015; Usami et al., 2015; Firrito et al., 2018). Nef and Vpu are also known to downregulate several tetraspanins such as CD81, CD63, and CD53, which are involved in the formation of tetraspanin-enriched microdomains (TEMs) (Haller et al., 2014). Nef further downregulates a plethora of cell surface receptors such as NKG2D-L required for NK cell activation. *In vitro*, it was shown that the decrease in levels of NKG2D-L that binds to NKG2D on NK cells reduced the cytolytic activity of co-cultured NK cells (Cerboni et al., 2007; Alsahafi et al., 2017). Apart from this, an essential aspect of Nef is that it also reduces the levels of MHC-I from the cell surface by using AP-1 to direct the MHCs to endosomes and lysosomes as a tactic of evading the immune response (Schwartz et al., 1996; Collins et al., 1998; Lubben et al., 2007). Thus HIV-1 accessory proteins, during binding, fusion, and budding, extensively remodel the plasma membrane and manipulate the host intracellular environment for productive infection and immune evasion.

## Summary

From entry to egress, at each step, HIV-1 depends on the host. This dependency also portrays the interaction with diverse cellular organelles that are otherwise essential for normal homeostasis. The plasma membrane is cleverly taken advantage of throughout the virus life cycle. Upon binding to HIV-1 gp120, various chemokine-dependent signal transduction pathways are rewired, many of which are crucial for immune effector functions. Further, the plasma membrane is the sight of HIV-1 budding, which is considerably reorganized to release newly formed virions. In addition to this, the cell surface protein composition is altered by accessory proteins like Nef and Vpu to counteract major host restriction factors, SERINC5 and Tetherin, respectively. The success of HIV-1 as a pathogen is perhaps imputed to these accessory proteins’ ability to hijack the host endocytic machinery and the trans-Golgi network efficiently to downregulate a vast number of cell surface proteins. Not only the interaction with endocytic machinery but Nef also utilizes the ER-associated protein degradation (ERAD) pathway for this purpose. After the efficient exploitation of the cell membrane, the viral core enters the cytoplasm where HIV-1 can interact with the cytoplasmic proteins and rearrange the cytoskeleton to sanction its retrograde transport toward the nucleus. Besides this, the viral CA can coherently interact with multiple host proteins to protect from premature uncoating and risking the viral genome being sensed by cytoplasmic immune sensors, and now we know that an intact capsid enters the nucleus. Apart from this, another viral accessory protein, Vif, can recruit the proteasomal machinery to degrade the host restriction factor APOBEC3G that induces mutations in the proviral DNA during reverse transcription resulting in truncated viral proteins or premature stop codons. After reaching the nuclear envelope, the viral core recruits host proteins like CPSF6 to employ nuclear importins, thus seizing the nuclear import machinery to transport into the nucleus. Within the nucleus, the virus becomes crucially dependent on nuclear proteins for uncoating and effective integration into actively transcribed regions of the host chromosome. Furthermore, the transcription of the integrated proviral DNA depends on the host RNA polymerase (RNA pol II). Although HIV-1 Tat considerably augments the transcription rate, it does so by interacting with host transcription factors like P-TEFB to utilize RNA pol II efficiently. After the generation of alternatively spliced and unspliced transcripts of HIV-1, they are transported to the cytoplasm for translation. The export of these viral transcripts is enabled by the viral protein Rev, which again depends on the host CRM1-dependent nuclear traffic. Once again, in the cytoplasm, HIV-1 employs the cellular protein translation machinery to produce viral proteins. Following this, the virus further takes advantage of host intracellular trafficking machinery for assembly of progeny near the plasma membrane, after which the virus buds off by altering the plasma membrane and recruiting the ESCRT machinery. Thus, the virus effortlessly exploits the machinery that is utilized by the host for its own survival and persistence.

## Concluding Remarks

The involvement of various subcellular entities in HIV-1 infection and their contribution to pathogenesis is becoming increasingly apparent. Thus, this review attempts to comprehend previously known and recently discovered compartmentalized cellular and molecular interactions during HIV-1 infection. With an increased understanding of host–virus cross talk, a future goal may be to utilize cutting-edge technologies, preferentially in relevant models, to identify candidates that could target organelle-specific host mechanisms. For instance, identifying how HIV-1 can evade innate sensors by preventing early uncoating, essentially to pathogenic effect, will have a profound impact on future drug developments. Consequently, a combination of therapeutic strategies in a fashion to abrogate compartmentalized interactions could prove to accentuate better adjunct treatment options.

## Author Contributions

PR, AKS, VB, and TM did literature review, generated figures using BioRender, and draft writing. AC conceived, reviewed, and edited the manuscript. All authors approved the final version.

## Conflict of Interest

The authors declare that the research was conducted in the absence of any commercial or financial relationships that could be construed as a potential conflict of interest.
